# High Expression of Stearoyl-CoA Desaturase 1 Predicts Poor Prognosis in Patients with Clear-Cell Renal Cell Carcinoma

**DOI:** 10.1371/journal.pone.0166231

**Published:** 2016-11-18

**Authors:** Jianfeng Wang, Yunze Xu, Liangsong Zhu, Yun Zou, Wen Kong, Baijun Dong, Jiwei Huang, Yonghui Chen, Wei Xue, Yiran Huang, Jin Zhang

**Affiliations:** Department of Urology, Renji Hospital, School of Medicine, Shanghai Jiaotong University, Shanghai, China; University of North Carolina at Chapel Hill School of Medicine, UNITED STATES

## Abstract

Stearoyl-CoA desaturase 1 (SCD1), the rate-limiting enzymes in the biosynthesis of monounsaturated fatty acids from saturated fatty acids, have been gradually recognized as a potential therapeutic target for various malignancies, particularly in clear-cell renal cell carcinoma (ccRCC). However, the prognostic value of SCD1 in ccRCC is still unknown. The aim of this study is to evaluate the clinical significance of SCD1 expression in patients with ccRCC. SCD1 expression in tumor tissues obtained from 359 patients who underwent nephrectomy for ccRCC are retrospectively assessed. During a median follow-up of 63 months (range: 1–144month), 56 patients in total died before the last follow-up in this study. Survival curves were plotted with the Kaplan–Meier method and compared with the log-rank test. Meanwhile, univariate and multivariate Cox regression models were applied to evaluate the prognostic value of SCD1 expression in overall survival (OS) for ccRCC patients. Moreover, SCD1 was enrolled into a newly built nomogram with factors selected by multivariate analysis, and the calibration was built to evaluate the predictive accuracy of nomogram. High SCD1 expression occurred in 61.6% (221/359) of ccRCC patients, which was significantly associated with age (p = 0.030), TNM stage (p = 0.021), pN stage (p = 0.014), Fuhrman grade (p = 0.014) and tumor sizes (p = 0.040). In multivariate analysis, SCD1 expression was confirmed as an adverse independent prognostic factor for OS. The prognostic accuracy of TNM stage, Fuhrman grade and tumor sizes was significantly increased when SCD1 expression was added. The independent prognostic factors, pT stage, pN stage, Fuhrman grade and tumor sizes, as well as SCD1 expression were integrated to establish a predictive nomogram with high predictive accuracy. Calibration curves revealed optimal consistency between observations and prognosis. In conclusion, high SCD1 expression is an independent prognostic factor for OS in patients with ccRCC. Our data suggest that the expression of SCD1 might guide the clinical decisions for patients with ccRCC.

## Introduction

Renal cell carcinoma (RCC) accounts for approximately 4% of all human malignancies, which is the most common primary cancer arising from kidney[1]. In 2016, it afflicts around 62700 new cases and causes nearly 14240 cancer-related deaths in The United States[2]. Although extraordinary progress in diagnosis and treatment of RCC has been achieved recently, the incidence rate of RCC is still increasing in a disturbing speed. Clear cell renal carcinoma (ccRCC) is by far the most common histology subtype of RCC, which accounts for over 80% of all kidney cancers[3]. Unlike most solid tumors, ccRCC has the innate chemotherapy- and radiotherapy-resistant nature, and nephrectomy can achieve a cure for early-staged ccRCCs, but there are still a high proportion of patients with metastases or advanced diseases present a dismal outcome and have limited therapeutic options[4]. Recently, for patients with metastatic ccRCC, the approved primary management was targeted therapies, which could remarkably improve patients’ prognosis [5]. However, targeted therapies have shown several obvious limitations, such as severe side effects and drug tolerance in the final process of therapies, and the management of metastatic ccRCC remains challenging[6, 7]. Thus, research must be conducted to investigate new biomarkers of ccRCC, which might provide new opportunities to identify potential prognostic factors and therapeutic targets for patients with ccRCC.

Saturated fatty acids (SFAs) and monounsaturated fatty acids (MUFAs) constitute the majority of the fatty acids in mammalian cells, and the ratio of MUFAs/SFAs is strictly regulated by cells since alterations in this balance can significantly change the physiological functions of cells[8]. Stearoyl-CoA desaturase (SCD), a central enzyme in lipid metabolism, catalyzes the biosynthesis of MUFAs from the saturated fatty acids (SFAs)[9]. Two isoforms of SCD (SCD1 and SCD5) were detected with different distribution in human tissues. Moreover, human SCD1, mainly distributed in adult adipose tissue, liver, lungs and brain, is an important regulatory enzyme to stimulate lipid biosynthesis to provide adequate phospholipids for cell membrane biogenesis and maintain normal signal transduction in the metabolism of cells[10, 11]. Recently, SCD1 has been extensively studied on cancer research and considered to be a novel molecular target for various tumors. Overexpression of SCD1 had been reported in many malignant cells, and upregulated levels of SCD1 activity have also been associated with the change of certain aspects of tumor cell behavior, such as tumor cell growth and proliferation [12, 13]. Furthermore, the prognostic significance of SCD1 expression was also revealed in many cancers, such as breast cancer, lung adenocarcinoma, colon cancer and soft tissue sarcomas, which showed that high expression of SCD1 was related to a poor outcome for patients with cancers[14–17]. Recent studies had also reported that SCD1 might be a novel molecular therapeutic target for ccRCC[18, 19]. As described in detail previously, SCD1 was overexpressed in human ccRCC tissues and ccRCC cell lines. It had also showed that knockdown of SCD1 gene expression in 786-O human ccRCC cells led to tumor cells apoptosis, significantly delays the formation of tumors, and reduces the growth rate of tumors formed[20]. However, the exact correlation between protein level and clinical significance of SCD1 expression in ccRCC patients still remains unclear.

In the present study, immunohistochemistry of tissues from patients with ccRCC were used to investigate SCD1 expression in tumor tissues, and correlations between SCD1 expression level and patient outcomes, as well as clinicopathological factors, were also investigated. Moreover, the nomogram integrating SCD1 expression and clinical features was also established to facilitate the study its potential utility as a prognostic marker and to enhance a better understanding of the role of SCD1 in ccRCC.

## Materials and Methods

### Patients

A total of 359 patients with ccRCC who underwent nephrectomy were collected from Renji Hospital of Shanghai Jiaotong University between Jan. 2001 and Dec. 2008. As a retrospective cohort study, consecutive patients are involved in, and all the tissue samples were obtained under consent of all the patients. Patients inclusion criteria for our study were as follows: (a) confirmed postoperative histopathology diagnosis as ccRCC; (b) all the patients had never received immunotherapy, chemotherapy, radiotherapy, or other antitumor therapy before and/or after surgery and (c)no comorbidities. Patients who are with mixed type RCC or familial RCC, lack of completed clinical and follow-up data were not included. For each patient, the following clinicopathological information were provided: age, gender, TNM stage, pT stage, pN stage, pM stage, Fuhrman grade and tumor size. TNM stage was confirmed under 2010 AJCC TNM classifications with radiographic reports and postoperative pathological results, and histological subtype of RCC was defined in accordiance with the 2014 EAU guidelines. Fuhrman grade was confirmed in line with 2012 ISUP consensus. Overall survival (OS) was defined as the interval from date of nephrectomy to the most recent follow-up or the day of death from any reason. Patients would be censored if they were still alive or if the patients were lost to follow-up. The last follow-up time was Apr. 30, 2012, and the median overall survival was 63 months (ranging from 1 to 144 months). This investigation was approved by the Ethics and Research Committees of Renji Hospital, Shanghai Jiao Tong University School of Medicine, and was conducted in accordance with the ethical standards and according to the Declaration of Helsinki and according to national and international guidelines. Tissue samples were obtained with written consent from all the patients.

### Immunohistochemistry

**Fig 1:**
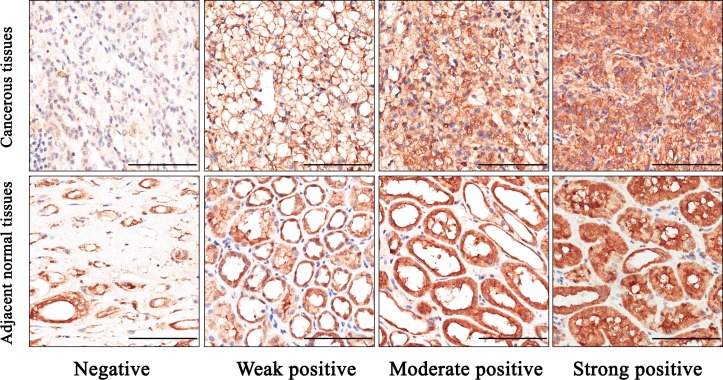
Representative immunohistochemical staining of SCD1 in ccRCC and adjacent normal tissues. **A.** Negative staining of SCD1 in ccRCC, score 0. **B.** Weak positive staining of SCD1 in ccRCC, score 3. **C.** Moderate positive staining of SCD1 in ccRCC, score 6. **D.** Strong positive staining of SCD1 in ccRCC, score 12. **E.** Negative staining of SCD1 in adjacent normal tissues, score 0. **F.** Weak positive staining of SCD1 in adjacent normal tissues, score 3. **G.** Moderate positive staining of SCD1 in adjacent normal tissues, score 6. **H.** Strong positive staining of SCD1 in adjacent normal tissues, score 12. Bar: 100 μm.

Immunohistochemistry was performed on tissue microarray (TMA) according to the standard streptavidin-peroxidase method (Zymed, San Francisco, CA, USA). Anti-SCD1 antibody (dilution 1:300; Abcam (ab19862), Cambridge, MA, USA) was used for immunohistochemistry staining. Meanwhile, antibody diluent (Abcam, ab64211) without primary antibody served as the control antibody. Results were recorded with Nikon eclipse Ti Microscope (Nikon Corporation, Tokyo, Japan) and Leica DM6000 B (Leica Microsystems, Wetzlar, Germany). The immunohistochemistry results were analyzed by two independent uropathologists who did not know the clinical condition of the patients. The expression level of SCD1 was scored by combining both the percentage of positive stained tumor cells and the staining intensity. As shown in Fig 1, the intensity of staining was scored as: 0 (negative), 1 (weak positive), 2 (moderate positive), or 3 (strong positive), and scores representing the percentage of positive cells was graded as: 1 (0–25%), 2 (26–50%), 3 (51–75%), or 4 (>75%). Comprehensive score = staining percentage × intensity. SCD1 expression level was classified as follows: <6, low expression; and ≥6, high expression.

### Statistical analysis

SPSS 19.0 software (SPSS Inc., IL, Chicago, USA) and R software version 3.1.2 with the “rms” package (R Foundation for Statistical Computing, Vienna, Austria) were used to perform statistical analysis in this study. χ2 test, Fishe’s exact method test and Cochran-Mantel-Haenszel χ2 test were applied for categorical data to analyze the correlation between SCD1 and clinicopathologic characteristics. Survival curves were plotted with Kaplan–Meier method and compared with the log-rank test. Significant variables were further analyzed by multivariate analysis to test independent prognosis. Harrell’s concordance index (C-index) and the Akaike information criterion (AIC) value were used to assess the predictive accuracy of different prognostic models. R software with “rms” package was used to generate the nomograms and calibration plots. Parameters statistically significant in multivariate analyses were selected to construct nomograms. In all cases, p<0.05 was considered statistically significant.

## Results

### Distribution of SCD1 in ccRCC

**Fig 2:**
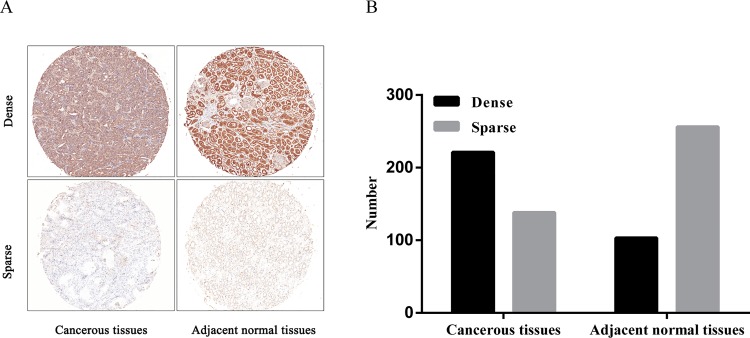
The distribution pattern and representative images of SCD1 High/low expression in tissues. **A.** Representative images of SCD1 staining with High/Low expression in ccRCC and adjacent normal tissues. **B.** The distribution pattern of SCD1 in ccRCC and adjacent normal tissues. Bar: 100 μm.

In order to investigate the role of SCD1 in ccRCC, firstly the SCD1 expression was evaluated by IHC analysis in TMAs among 359 ccRCC patients. As shown in Fig 2A, SCD1 was detected both in cancerous tissues and paired adjacent normal tissues, with variable staining intensity in different specimens. Moreover, there was a significant difference in the extent of SCD1 staining between cancerous and paired adjacent normal tissues (Fig 2B and Table 1; p < 0.001). SCD1 was found to be overexpressed in ccRCC tissues with high expression rate of 61.6% (221/359). In contrast, the expression of SCD1 was greatly inhibited in adjacent normal tissues with high expression in 28.7% (103/359) of cases. In brief, the expression of SCD1 was higher in ccRCC tissues than in adjacent normal tissues (p<0.001)

**Table 1:** Comparisons with SCD1 expression between ccRCC and paired adjacent normal tissues.

		Expression of SCD1 (n, %)		
Tissue sample	No.of patients			*P*-value
		Low	high	
Tumor tissues	359	138(38.4%)	221(61.6%)	
				p<0.001*
Adjacent normal tissues	359	256(71.3%)	103(28.7%)	

*p<0.05 was considered statistically significant.

### Relationship between clinicopathological features and SCD1 expression in ccRCC patients

Totally,359 patients [254 men (70.75%) and 103 women (29.25%)] aged between 15 and 85 years (median 55.78 years) were included in this study as shown in Table 2. Tumor size ranged from 1.0 to 14.0 cm with a median of 1.25 cm, and 3-year survival rate was 94.99% (341/359). Among all cases, the patients distributions of TNM stage I, II, III, and IV were 287, 55, 10, and 7, accounting for 79.94%, 15.32%, 2.79%, and 1.95% respectively. Moreover, the patients proportions of Fuhrman grades 1, 2, 3, and 4 were separately 32.59% (117), 50.14% (180), 15.60% (56), and 1.67% (6). In all cases, lymph node metastasis was presented in 9 patients, while 6 patients were observed with distant metastasis at the time of surgery.

**Table 2:** Detailed clinical information and follow-up data of 359 patients with ccRCC.

Characteristics	Categories	Number
Overal survival median (range, months)		63.01(1–144)
Age median (range, years)		55.78(15–85)
Tumor size median (range, cm)		1.25(1–14)
Gender		
	Male	254
	Female	103
TNM stage		
	T1	287
	T2	55
	T3	10
	T4	7
pT stage		
	T1	289
	T2	56
	T3	10
	T4	4
pN stage		
	N0(negative)	350
	N1(positive)	9
pM stage		
	M0(Absent)	353
	M1(Present)	6
Fuhrman grade		
	I	117
	II	180
	III	56
	IV	6
Survival outcome		
	Dead	56
	Survival or lost	303
3-year survival rate(number)		94.99%(341/359)

Then, the association of those clinicopathological variables with the SCD1 expression level were analyzed. As summarized in Table 3, the expression of SCD1 in cancerous tissues was significantly related to age (p = 0.030), TNM stage (p = 0.021), pN stage (p = 0.014), Fuhrman grade (p = 0.014) and tumor sizes (p = 0.040) for patients with RCC, whereas it was not associated with patients’ gender, pT stage, pM stage (p>0.05).

**Table 3:** Association of SCD1 expression with clinicopathological characteristics in ccRCC patients.

Characteristics		Patients		Tumoral SCD1 expression		
		n	%	Low	high	*P*-value
All patients		359	100	138	221	
Gender						1.000^a^
	Male	254	70.8	98	156	
	Female	105	29.2	40	65	
Age(years)						0.030^a^*
	≤55	179	49.9	79	100	
	>55	180	50.1	59	121	
TNM stage						0.021^b^*
	I+II	342	95.3	136	206	
	III+IV	17	4.7	2	15	
pT stage						0.090^b^
	T1+T2	345	96.1	136	209	
	T3+T4	14	3.9	2	12	
pN stage						0.014^b^*
	N0	350	97.5	138	212	
	N1	9	2.5	0	9	
pM stage						0.413^b^
	M0	353	98.3	137	216	
	M1	6	1.7	1	5	
Fuhrman grade						0.014^a^*
	I+II	297	82.7	123	174	
	III+IV	62	17.3	15	47	
Tumor size(cm)						0.040^a^*
	≤4	186	51.8	81	105	
	>5	173	48.2	57	116	

^a^Chi-square test.

^b^Fisher’s exact test.

*P<0.05 indicates a significant association among the variables.

### Associations between SCD1 expression and patient survival

**Fig 3:**
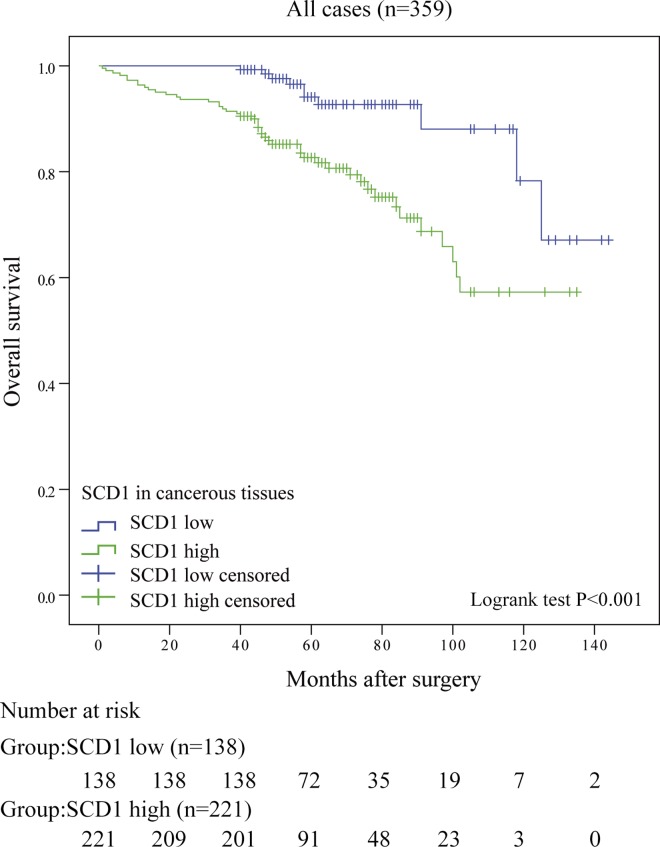
Overall survival curves based on SCD1 expression in ccRCC tissues. Kaplan-Meier analysis of OS, P value was calculated by log-rank test.

Kaplan–Meier survival curves and log-rank tests were performed to investigate the association between SCD1 and the overall survival (OS) of ccRCC patients. As presented in Fig 3, patients with high SCD1 group had a worse OS (*P*<0.001) than those in low SCD1 group. Further, univariate and multivariate analyses were undertook to make sure whether SCD1 is an independent predictor for ccRCC prognosis. In univariate analysis, SCD1 expression was found to be significantly associated with the overall survival of ccRCC patients (p<0.001, Table 4). Moreover, Univariate analysis also indicated that TNM stage, pT stage, pN stage, pM stage, Fuhrman grade and tumor sizes were correlated significantly with patient survival (all p<0.001; Table 4). Next those parameters were put in multivariate analysis, confirming that SCD1 expression was still predictable for ccRCC outcomes (p = 0.003). Together with SCD1, pT stage (p = 0.019), pN stage (p = 0.028) Fuhrman grade (p<0.001) and tumor sizes (p = 0.003) were all considered as independent predictors of OS for RCC patients (Table 4).

**Table 4:** Summary of univariate and multivariate Cox regression analysis of overall survival duration in all ccRCCs.

Variables		Univariate analysis			Multivariate analysis		
		HR	(95% CI)	p*	HR	(95% CI)	p*
SCD1 in cancer tissues							
	Low	1			1		
	High	3.623	1.817–7.221	P<0.001*	2.944	1.439–6.026	0.003*
SCD1 in noncancer tissues							
	Low	1					
	High	0.673	0.355–1.276	0.225			
Gender							
	Male	1					
	Female	0.849	0.470–1.535	0.589			
Age(years)							
	≤55	1					
	>55	1.359	0.800–2.310	0.257			
TNM stage							
	I+II	1			1		
	III+IV	9.551	4.957–18.403	<0.001*	0.353	0.039–3.177	0.353
pT stage							
	T1+T2	1			1		
	T3+T4	12.993	6.605–25.558	<0.001*	12.265	1.512–99.485	0.019*
pN stage							
	N0	1			1		
	N1	15.775	7.308–34.054	<0.001*	4.140	1.166–14.694	0.028*
pM stage							
	M0	1			1		
	M1	8.908	3.170–25.036	<0.001*	1.729	0.444–6.726	0.430
Fuhrman grade							
	I+II	1			1		
	III+IV	3.897	2.293–6.624	<0.001*	3.134	1.776–5.531	<0.001*
Tumor size(cm)							
	≤4	1			1		
	>5	5.907	2.789–12.511	<0.001*	3.331	1.517–7.311	0.003*

*p<0.05 was considered statistically significant.

### Comparison of the predictive abilities between SCD1 expression and other prognostic factors

In order to further confirm the predictive ability of SCD1 expression, SCD1 expression was compared with conventional prognostic factors like TNM staging system, Fuhrman grade and tumor sizes, respectively. Harrell concordance index (*C*-index) and Akaike information criteria (AIC) analysis were applied to investigate the prognostic accuracy. As presented in Table 5, the *C-*indexes of SCD1 expression for OS was 0.648, while TNM stage, Fuhrman grade and tumor sizes were 0.610, 0.695, and 0.687 respectively. Noticeably, the *C-*index of those models was improved to 0.714, 0.755, and 0.749 respectively when SCD1 expression signature was replenished for OS. Besides, the AIC values presented among all combined models were lower than their conventional model alone.

**Table 5:** Comparison of the predictive accuracies of conventional prognostic factors.

Model	Overall Survival(N = 359)	
	C-Index	AIC
SCD1	0.648	566.497
TNM2	0.610	553.538
SCD1+TNM2	0.714	542.431
Fuhrman grade	0.695	554.645
Fuhrman grade+SCD1	0.755	543.276
Tumor size	0.687	552.600
Tumor size+SCD1	0.749	541.425
Nomogram	0.812	513.931

C-index, Harrell’s concordance index; AIC, Akaike information criteria.

### Prognostic nomogram for OS of patients with ccRCC

**Fig 4:**
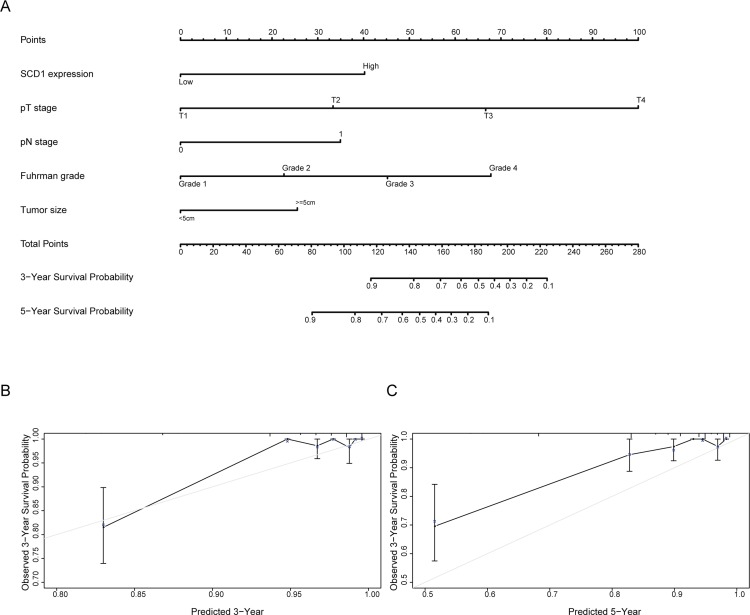
Nomogram and calibration plot for prognosis of OS in patients with ccRCC. **A.** Postoperative prognostic nomogram of patients with ccRCC. **B.** The calibration plots for overall survival at 3 years. **C.** The calibration plots for overall survival at 5 years.

In order to use SCD1 as a prognostic parameter, nomogram was built for OS (Fig 4A) in ccRCC patients via integrating all the independent prognostic indicators in the light of the results of multivariate analysis. Patient survival probabilities at different time after surgery could be predicted by total points which were calculated by adding up each point of each parameter. The calibration plot for the probability of OS at 3 or 5 year after surgery revealed good consistency between actual observation and the prediction by nomograms. (Fig 4B and 4C).

## Discussion

Currently, outcome prediction in ccRCC is based on several established prognostic systems and algorithms, insufficient for accurately predicting survival of patients with ccRCC, for which targeting therapy is the primary decision to make in patient management [21, 22]. Thus, a new biomarker to improve the current prognostic systems and provide a novel molecular therapeutic target is in desperate need for ccRCC patients. This study showed that there was a notable association between high SCD1 expression and poor outcome in patients with ccRCC. Moreover, SCD1 expression has also been discovered as an independent prognostic factor and could be used to construct a nomogram with acknowledged pathologic factors. In addition, higher SCD1 levels in patients with lymph node metastasis and high Fuhrman grade were observed, which suggested that SCD1 might play a vital role in the metastasis of ccRCC.

Recently, alterations of lipid metabolism had been increasingly recognized as a central feature of cancer cells, which is mainly manifesting as the excessive synthesis of fatty acid [23]. Fatty acids are the most important components of cellular structure and play a vital role in cell normal function, such as participating in the synthesis of cell membrane, presenting as precursors and lipid second messenger and as anchor structural protein fixed on the membrane[24]. Cancer transformed cells, characterized by their ability to divide more frequently without immortality, the invasive ability to adjacent normal tissues and the loss of contact inhibition, compared with normal cells. Therefore, in order to adapt to cancer cells progressions, cancer cells need to coordinate the activation of lipid biosynthesis and the signaling networks that stimulate this process[8]. In the present study, we have discovered that SCD1 was upregulated which may be used as an anti-cancer target for ccRCC. SCD1, a critical regulator of energy metabolism and catalyzes the synthesis of monounsaturated fats, plays an essential role in maintaining the balance of the ratio of SFA/MUFA which would promote cellular lipid synthesis and meet the functional requirements of cells[25]. Recent researches showed that kidney is not a single disease but encompasses a number of different types of cancer that occur in the kidney. The major mutations in kidney cancer genes result in dysregulation of metabolic pathways involved in oxygen, iron, energy and/or nutrient sensing suggesting that kidney cancer is a disease of cell metabolism[26]. Therefore, the dysregulation of lipid metabolism may also participate in the progress of ccRCC.

Although SCD1 is gradually recognized as a prognostic biomarker in many malignancies, the exact functional role that SCD1 plays in ccRCC is still blurred. In the present study, we discovered that low expression level of SCD1 predicted a longer survival time of ccRCC patients. Similar to our study, Holder et al suggested that high levels of SCD1 expression are associated with significantly shorter RFS and OS in breast cancer. Furthermore, they showed that SCD1 expression varies by breast cancer subtype [14]. Huang et al reported that the pathological stage in patients with lung adenocarcinoma was associated with the SCD1 mutation, which is associated with poor prognosis. During the study, they observed that high expression of SCD1 in lung adenocarcinoma was required for the cell proliferation, migration and invasion, which suggested that high expression of SCD1 remarkably enhanced the ability of tumor formation and invasion[15]. However, the exact mechanism that how SCD1 work in the tumorigenesis and development in ccRCC is remaining vague. Von Roemeling et al reported that SCD1 might be a novel molecular therapeutic target for clear cell renal cell carcinoma, for which increased SCD1 expression was observed in ccRCC and it was essentially associated with ccRCC viability[19]. As showed in our previous study, there might be positive feedback loop and synergistic effects between SCD1 and hypoxia-inducible factor-2α (HIF-2α), which suggested that SCD1 might have a cross-talk with HIF in ccRCC[20]. Anyway, further studies need to be carried out in ccRCC to decipher the mechanism of aberrant SCD1 upregulation.

Several limitations of this study should be acknowledged. Although we have concluded and presented some novel findings about SCD1 expression in ccRCC, more efforts need to be exerted in future studies. First of all, the conclusion we made may not be credible for there was a single-center study. Thus, more researches with multicenter patients will be needed for further investigation of the specific role of SCD1 in ccRCC. Another problem is that, although we have analyzed a considerable number of patients with ccRCC, patients with ccRCC of TNM stage III or IV seem to be insufficient in the study. In addition, the follow-up time of patients in the study may not be persuasive for illustrating the relationship between SCD1 and patients’ survival, which calls for longer time of follow-up to clarify their relation. Nevertheless, we have provided evidence indicating that SCD1 is over-expressed in ccRCC and is associated with a poor outcome for patients. What’s more, our data suggested that SCD1 might be of a pivotal role in the progression of ccRCC and could be integrated to the current model to predict survival of ccRCC as an independent prognosis factor, which might guide the clinical therapies. However, much efforts in exploring the relationship between SCD1 and ccRCC is required in future studies.

In conclusion, the present study investigated the expression pattern and clinicopathological significance of SCD1 in ccRCC and showed that SCD1 might be of a pivotal role in the progression of ccRCC and could be integrated to the current model as an independent prognosis factor to predict survival of ccRCC, which might guide the clinical decisions. However, the clinical role of SCD1 in ccRCC is merely investigated and its specific mechanisms and effects in vitro still need further research.

## Supporting Information

S1 Fig Overall survival curves based on SCD1 expression in cancerous and adjacent tissues. Kaplan-Meier analysis of OS, P value was calculated by log-rank test.
(PDF)

S1 Table Comparisons with SCD1 expression between cancerous tissues and paired adjacent normal tissues. (DOCX)
