# Supplementary figures and images for: High Expression of Stearoyl-CoA Desaturase 1 Predicts Poor Prognosis in Patients with Clear-Cell Renal Cell Carcinoma
Source: PLoS One. 2016 Nov 18;11(11):e0166231. doi: 10.1371/journal.pone.0166231 (PMC5115711; doi:10.1371/journal.pone.0166231)

S1 Fig. Overall survival curves based on SCD1 expression in cancerous and adjacent tissues

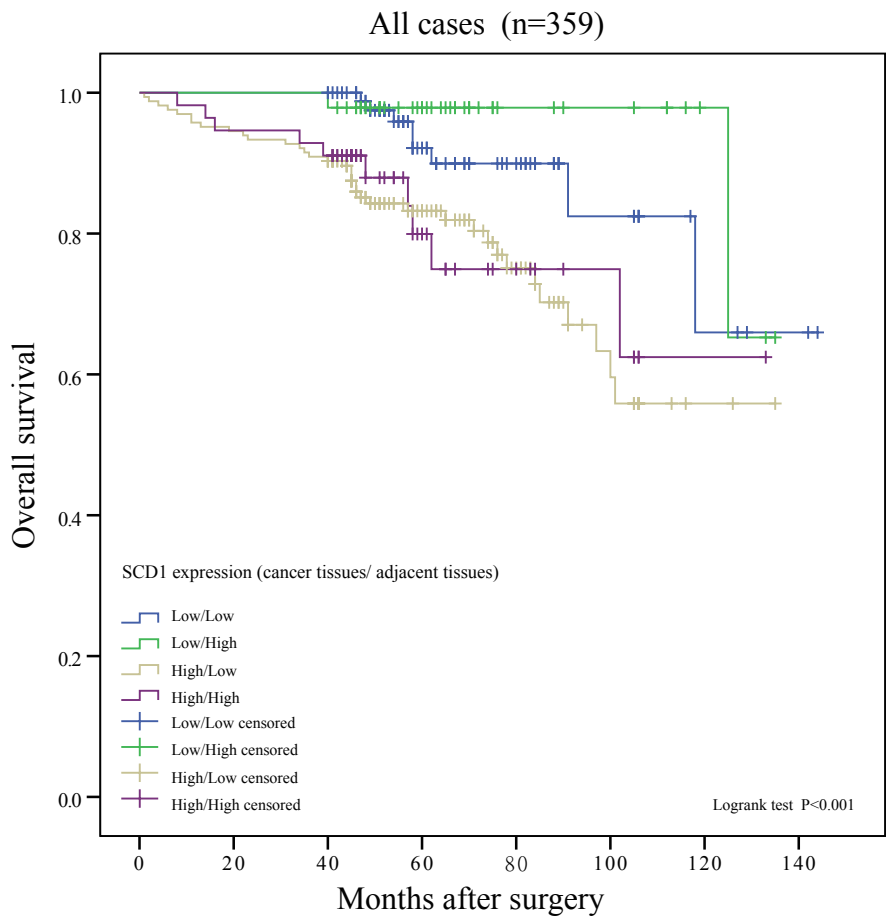

Supplement: S1 Fig — Kaplan-Meier analysis of OS, P value was calculated by log-rank test. (PDF) [file pone.0166231.s001.pdf]
