# Supplementary material for: High Expression of Stearoyl-CoA Desaturase 1 Predicts Poor Prognosis in Patients with Clear-Cell Renal Cell Carcinoma
Source: PLoS One. 2016 Nov 18;11(11):e0166231. doi: 10.1371/journal.pone.0166231 (PMC5115711; doi:10.1371/journal.pone.0166231)
Supplement: S1 Table — (DOCX) [file pone.0166231.s002.docx]

| **S1 Table: Comparisons with SCD1 expression between cancerous tissues and paired adjacent normal tissues.** | | | | | | |
| --- | --- | --- | --- | --- | --- | --- |
|  |  | Expression of SCD1 in cancerous tissues (n, %) | | |  |  |
|  |  | Low |  | High |  | *P*-value |
|  |  |  |  |  |  |  |
| SCD1 in Adjacent  normal tissues | Low | 91(25.3%) |  | 165(46.0%) |  |  |
|  |  |  |  |  |  | p<0.001* |
|  | High | 47(13.1%) |  | 56(15.6%) |  |  |

*p<0.05 was considered statistically significant.
